# Performance Evaluation of Energy-Autonomous Sensors Using Power-Harvesting Beacons for Environmental Monitoring in Internet of Things (IoT)

**DOI:** 10.3390/s18061709

**Published:** 2018-05-25

**Authors:** George Dan Moiş, Teodora Sanislav, Silviu Corneliu Folea, Sherali Zeadally

**Affiliations:** 1 Department of Automation, Faculty of Automation and Computer Science, Technical University of Cluj-Napoca, 400114 Cluj-Napoca, Romania; Teodora.Sanislav@aut.utcluj.ro (T.S.); Silviu.Folea@aut.utcluj.ro (S.C.F.); 2 College of Communication and Information at the University of Kentucky, Lexington, KY 40506-0224, USA; szeadally@uky.edu

**Keywords:** air quality, Bluetooth Low Energy, environmental monitoring, low-power electronics, energy Harvesting

## Abstract

Environmental conditions and air quality monitoring have become crucial today due to the undeniable changes of the climate and accelerated urbanization. To efficiently monitor environmental parameters such as temperature, humidity, and the levels of pollutants, such as fine particulate matter (PM2.5) and volatile organic compounds (VOCs) in the air, and to collect data covering vast geographical areas, the development of cheap energy-autonomous sensors for large scale deployment and fine-grained data acquisition is required. Rapid advances in electronics and communication technologies along with the emergence of paradigms such as Cyber-Physical Systems (CPSs) and the Internet of Things (IoT) have led to the development of low-cost sensor devices that can operate unattended for long periods of time and communicate using wired or wireless connections through the Internet. We investigate the energy efficiency of an environmental monitoring system based on Bluetooth Low Energy (BLE) beacons that operate in the IoT environment. The beacons developed measure the temperature, the relative humidity, the light intensity, and the CO_2_ and VOC levels in the air. Based on our analysis we have developed efficient sleep scheduling algorithms that allow the sensor nodes developed to operate autonomously without requiring the replacement of the power supply. The experimental results show that low-power sensors communicating using BLE technology can operate autonomously (from the energy perspective) in applications that monitor the environment or the air quality in indoor or outdoor settings.

## 1. Introduction

Environmental monitoring is an important and highly active research area. The observation of current values and trends of environmental parameters, such as temperature or level of harmful gases in the air, provides data that can help the detection of hazardous events and the assessment and implementation of appropriate actions in the case of climate change, population growth, urban sprawl, invasive species, and habitat destruction [1]. There is a close correlation between environmental pollution, human health and socioeconomic development as pointed out in a recent World Health Organization report which states that in 2012 one in eight deaths globally was caused by air pollution exposure [2,3]. The air quality index (AQI) has therefore been defined by government authorities for quantifying the magnitude of air pollution. Although different countries specify their own indices, the overall value is computed based on the concentration of different air pollutants such as PM_2.5_ and PM_10_ particles, and SO_2_, NO_2_, CO, and O_3_ chemical compounds. Another phenomenon with severe consequences, which is influenced by the continuously increasing impact of human activities over the environment, with carbon dioxide emissions as a primary cause, is climate change [4,5]. These threats can be addressed by selecting appropriate adaptation and mitigation options that have sustainable development as an ultimate goal. In this context, both individuals and public authorities have to take actions based on the relevant information extracted from huge amounts of data provided by environmental monitoring applications whose development has become a priority [6]. The work presented in this paper aims to achieve energy-efficient autonomous operation of air quality sensors, and represents an initial step in the development of scalable low-cost systems based on wireless sensors for the fine-grained evaluation of environmental conditions and of the quality of the air in indoor and outdoor places. The analysis of the operation and power consumption of the wireless air quality sensors developed has led to the development of optimized firmware that enables energy autonomy.

In the vast majority of cases, monitoring applications depend on the deployment of large networks of radio sensors, grouped into wireless sensor networks (WSNs). WSNs enable the implementation of various application objectives within specific sensing fields, mainly because of their increased flexibility, scalability, and reduced installation and maintenance costs [7]. WSNs consist of large numbers of resource-constrained sensors that possess processing and wireless communication capabilities, and their main goal is the delivery of sensed data to a base station [8]. The sensors in WSNs, called nodes or motes, have three basic functionalities, which include sensing, data processing, and communication, and consist of four main components: a sensing unit, a processor, a transceiver, and the power supply [8]. Large numbers of low-cost sensors are used for covering vast areas, and in many cases they are placed in remote and hard to reach locations [9]. This makes the use of power cables or batteries infeasible, due to the efforts required for commissioning (installing cables) and maintenance (changing batteries). As a result, low-power operation represents one of the major challenges this type of system faces as reported in the literature which highlights various solutions to address energy usage issues in WSNs [10]. Some of the solutions focused on the development of energy-efficient communication protocols [11,12], on sleep-scheduling [13], or on harvesting energy from the environment [14,15,16] for prolonging the operation time of motes and the lifetime of entire WSNs.

By providing sensors with the capability of sending the sensed data over the Internet, through the use of compatible technologies and standards (e.g., WI-Fi, IEEE 802.11) or through the use of gateways for packaging the information in Internet-based protocols (e.g., UDP, HTTP), they become “smart objects” belonging to the Internet of Things (IoT) vision [17]. This vision imagines a world in which “people and things” are “connected Anytime, Anyplace, with Anything and Anyone, ideally using Any path/network and Any service” [18]. The implementation of such communication mechanisms is more feasible in urban areas or in indoor places where a wide rage of connectivity options to networks having native Internet compatibility are prevalent. These are also the places where air quality is a major concern because of the presence of high concentrations of gaseous pollutants such as NO_2_, NO, SO_2_, CO that are harmful to humans and whose values have to be monitored and kept below established limits. Volatile organic compounds (VOCs) are also significant environment pollutants and are a threat to human health because they consist of toxic chemicals that can cause irritations, headaches or can even damage the central nervous system [19,20].

The use of gas sensors (such as wireless sensors in WSNs) in energy-constrained systems opens up challenges such as high power consumption that needs to be addressed. However, recent advances in nanotechnology and microelectromechanical systems (MEMS) have made the development of increasingly powerful and energy-efficient designs [21,22] possible with state-of-the art gas sensors. Devices such as the COZIR AMB CO_2_ Sensor from Gas Sensing Solutions [23], CCS811 produced by ams [24] and BME680 from Bosch Sensortec [25] are now available on the market thereby facilitating the production of cheap, low-power systems for environmental monitoring or for air quality evaluation. This work analyzes and optimizes the power consumption and energy efficiency of a Bluetooth Low Energy (BLE) air quality sensor that broadcasts the acquired data using advertisements packets. The wireless sensor we have developed is equipped with power harvesting capabilities and incorporates the CCS811 which is capable of measuring the total volatile organic compounds (TVOC) concentration value and equivalent CO_2_ (eCO_2_) levels. The wireless sensor consists of a Cypress EZ-PRoC BLE module [26], which includes a 32-bit processor operating at up to 48 MHz and a BLE transceiver for the processing and communication units, the CCS811 (eCO_2_ and TVOC), the SHT21 (temperature and relative humidity) [27] and the OPT3001 [28] (light intensity) integrated circuits (ICs) as the perception component, and a power management circuit, an accumulator and two amorphous silicon solar cells as the power supply.

We summarize the main contributions of the paper as follows:

- We developed adaptive duty-cycling strategies (self-adapting strategies for achieving autonomous operation from an energy perspective) to optimize the operation of environmental sensors such as gas sensors.
- We achieved energy-efficient autonomous operation of wireless sensors that measure the temperature, relative humidity, light intensity, and equivalent CO_2_ and TVOC levels.
- We analyze the operation of a BLE sensor for environmental monitoring that can measure T, RH, equivalent CO_2_ and TVOC concentrations, and light intensity.
- We have designed a complete IoT-based solution for monitoring temperature, relative humidity and equivalent CO_2_ and VOC concentration levels within a large area.
- We developed a monitoring system that can be used in the air quality mapping of buildings or open areas.

The remainder of the paper is organized as follows. Section 2 presents the related works that focus on energy-autonomous wireless sensors for environmental or ambient monitoring, while Section 3 describes our proposed solution. Section 4 presents the power consumption of the developed device when different energy consumption strategies are used. Section 5 presents the empirical results obtained and finally, the conclusion and the future work are outlined in the last section.

## 2. Related Work

Significant research efforts have been carried out for achieving energy-efficient electronic devices capable of measuring the concentration of different pollutants in the air by monitoring applications and for evaluating the different approaches that were adopted [22,29,30,31,32,33,34]. We describe some of the most important works in this section.

### 2.1. Air Quality Monitoring Platforms

Kim et. al. [29] discuss the issues, infrastructure, data processing, and the challenges related to the design and implementation of an integrated sensing application capable of detecting the level of seven gases (ozone, particulate matter, carbon monoxide, nitrogen oxides, sulfur dioxide, volatile organic compound, and carbon dioxide) in indoor spaces. The sensing system they designed consists of a sensor network cloud (SNC) and a sink node that relays the data to the middleware where it is stored and interpreted. The sensor nodes are based on the Raspberry Pi single-board computer which runs an algorithm that smooths and aggregates the data acquired from the attached sensors and adjusts it based on the readings from a relative humidity and temperature transducer. The connection to the cloud or to the Internet of Things framework is established through low-power radio modules operating in the 2.4 GHz industrial, scientific and medical (ISM) band. The information is received by the sink node which is connected to the middleware through a wired serial connection. The architecture of the system enables increased measurement accuracy, efficient auto-calibration mechanisms, and reduced traffic and energy savings for communication operations. Although the sensing device presented is able to monitor a wide range of pollutant gases and its use could be extended for the outdoor environment or for additional compounds, its complexity and consumption prevents it from being powered from batteries or from energy harvesting modules.

The work in [30] is concerned with the development of an end-to-end distributed monitoring system for the detection of VOC and hydrogen sulfide (H_2_S) emissions in hazardous environments. The developed system consists of node sub-networks positioned in locations that are critical to a plant, and each node is connected to a gateway that provides Internet access. The architecture implements a typical IoT scenario wherein the data gathered every minute is sent to a central station, from where it can be accessed by authenticated users. The gateways are called sink node units (SNUs) and have the capability of measuring the temperature, humidity and wind speed of the air using the attached sensors. These also forward the data received from the wireless end node units (ENUs) connected to them every minute to the central server. Next, the ENUs are fitted with VOC and H_2_S detectors through which they communicate using an RS485 serial connection. However, because of the high energy demand of the peripherals, especially the VOC sensors, the ENUs use two different power supplies, one for the microcontroller and one for the detectors. For assuring energy autonomy, all the components in the system are equipped with photovoltaic panels as a secondary source that provide energy for operation and for charging the onboard batteries. The system has been successfully tested at two different industrial sites, and continuous unattended operation, even without battery replacements has been achieved.

Velasco et. al. present a mobile wireless sensor system that can measure the level of PM_10_ and O_3_ in the environment in the Turin area, a critical zone in the European Union regarding air pollution [31]. The system they developed represents a cheaper alternative for scientific air quality monitoring equipment and can complement the readings of official measuring stations. It can significantly extend the supervised area and help in the achievement of a fine-grained representation of the pollutant concentrations in the air. For doing this, it relies on mobile nodes equipped with wireless communication capabilities attached to bicycles belonging to the public bike sharing system. The data can be collected by stationary hubs or gateways that forward it to a central server for further filtering and processing. The “Waspmote Plug & Sense” platform was selected as the sensor node in the proposed monitoring application because of its reduced cost, potability and reliability. The module communicates using ZigBee technology, offering a satisfactory trade-off between packet loss ratio, range, and power consumption. Although in this configuration the module drains the battery in less time than when using the Wi-Fi or Bluetooth Low Energy technologies, it can operate uninterrupted during the bicycle trips and can be recharged at the bike docking stations. The tests performed show that the proposed system is capable of covering an extended area and that it provides air quality information that can be used for complementing official measurements thereby achieving street-level resolution despite the fact that it outputs less accurate data.

The idea of using citizens as sensor carriers or as entities that can be viewed as “human sensors” to some degree for obtaining fine-grained ambient air quality information has also been studied. The authors of [32] propose a system for the monitoring of urban air quality that relies on participatory sensing, a concept that assumes the collection of data regarding specific environmental parameters such as temperature, humidity, and PM_2.5_, from individuals and communities by using sensor enhanced smartphones and cloud services. The sensors in this approach can be built into the communication device, can be connected to it using cables or wireless channels (USB connected off-the-shelf PMS5003ST sensor for temperature, humidity and particle concentration), or can consist in the persons themselves, rating the quality of the air using a predefined metric. The metric consists of several levels, starting from *comfortable*, continuing with *acceptable*, *lightly allergic*, and *allergic uncomfortable*, and ending with *very uncomfortable*. As can be seen, instead of focusing on acquiring highly accurate data, the system encourages the people to get involved in sensing tasks and for providing information useful in environmental protection. To minimize the power required by the applications running on the participants’ smartphones, the authors propose AS-air (Adaptive Sampling Scheme for Urban Air Quality), an energy-efficient adaptive sampling scheme, that modifies the data acquisition rate depending on the environment, on historical data, and on the running platform’s available power budget. An evaluation of the proposed solution showed that it is able to provide a sampling scheme that leads to power savings and higher performance in terms of adaptivity in comparison with the traditional Q-learning scheme.

The authors of [22] developed a modular end-to-end indoor air quality monitoring (IAQM) system based on a wireless sensor network that provides data regarding the CO_2_, CO, SO_2_, NO_2_, O_2_, Cl_2_, temperature, and relative humidity. The result of the research work is a complete solution that includes gateways for gathering the acquired data and an IoT server that disseminates the data to remote users who can visualize it in graphical or tabular form. The sensor nodes are based on the Libelium platform that represents the processing module, an XBee PRO module as the communication part, and seven sensors. The network uses a star architecture. The sensor nodes sending the data to the gateways use single hop communication. The nodes are powered from the main lines while the on-board accumulator of 6600 mAh maintains the real-time clock in case of temporary power failure. They are also provided with a back-up tool for storing the data when communication links are interrupted. A Raspberri Pi single-board computer is programmed through Python scripts for implementing the core of the gateway. Its two main tasks consist of relaying the data received from the sensors to the Emoncms web-server and recovering packets that were lost during communication. The system has been validated in a real scenario where it monitored the air quality within a university campus and it will be deployed in various other locations in Doha-QUATAR.

There are also commercial solutions such as BOSCH’s “Plantect^TM^” [35] or the SensorInsight Air Quality Index Solution Kit from Libelium [36] that rely on ambient monitoring sensors which are available on the market. The first one is a disease prediction system for greenhouse grown tomatoes based on Artificial Intelligence and on light intensity, temperature, humidity, and carbon dioxide sensors, while the second is a kit for the real time monitoring of air quality in cities, that includes probes for measuring the levels of pollutants, such as carbon dioxide, methane, carbon monoxide, and others.

As can be seen from the literature review, many recent research efforts have focused on fine-grained readings even if the values are not as accurate as the ones provided by official measuring stations. This shortcoming is compensated by the reduced form factor, low costs and modularity of the proposed approaches. However, several of the solutions presented include power-hungry gas sensors and have to be connected to the power lines. Therefore, for gaining wider acceptance and for overcoming the power consumption problem, new approaches have to take advantage of the features provided by newly developed low-power sensors as well as techniques such as energy harvesting. Due to the components that we used and to the algorithms implemented in firmware, the wireless sensor we have developed has a power consumption that allows it to be powered by a small capacity accumulator charged by two small factor solar panels (22 mm × 7 mm each). This led to the development of an autonomous sensing system with dimensions of only 35 mm × 35 mm.

### 2.2. Energy Harvesting Solutions

Energy harvesting or energy scavenging is the process of capturing energy from external power sources, such as vibration, solar, heat, electromagnetic waves, and others. It is used for extending the battery lifetime of electronic systems and in the case of low power electronic devices, to completely replace them. Given the energy constraint associated with wireless sensor nodes in general and in particular gas sensing applications, some of the approaches propose designs that include energy harvesting mechanisms [37,38,39].

The authors of [37] propose an energy aware Adaptive Sampling Algorithm for WSNs that complements solar energy harvesting by modifying the sampling period of the attached sensors depending on the available energy. The system was validated through the in-field evaluation of a ZigBee network that monitors the bees in a beehive. This includes power hungry nodes to which off-the-shelf gas sensors that measure the carbon dioxide, oxygen, nitrogen dioxide, and air contaminants levels, are connected. The results show that energy harvesting plays a major role in achieving self-sustainability when appropriate adaptive sampling techniques are implemented.

The work in [38] is concerned with the development of a ZigBee-based Wireless Gas Sensing Network (WGSN) that makes use of adaptive sleep cycles and solar energy harvesting mechanisms for extending its lifetime. Te system can be used for detecting the presence of toxic and combustible gases in the Oil and Gas industry. The nodes can sense the level of methane, hydrogen sulfide, and carbon monoxide through the attached metal oxide semiconductor gas sensors. The proposed system was tested using modelled gas behaviour, and the results obtained show that energy harvesting can significantly increase the life time of the system, reaching a period of 5.5 years. Furthermore, network operation could be extended by a cooling system and by the automatic cleaning of the solar panel.

Wu et. al. [39] developed a wireless sensor network that can monitor the temperature, humidity, carbon monoxide, and carbon dioxide levels, while being powered by a 55 mm × 67.5 mm solar panel. The sensor node includes an XBee module for performing communication tasks, a microcontroller as the data processing part, and four sensors (MCP9700 temperature sensor, HIH5030 humidity sensor, MiCS-5121WP CO sensor, and COZIR^TM^GC-0012 CO_2_ sensor). The system is a duty-cycled one, spending 15 s in the wake-up mode and 20 min in the sleep state. The experiments performed demonstrated that in the case of systems that include large-capacity rechargeable batteries the proposed architecture and firmware lead to a solution that can keep the wireless nodes active and reliable for the duration of an entire day.

The literature indicates that solar power is the preferred option when implementing energy harvesting schemes mainly because it is a mature technology [40] and also because of the power density it provides which is larger than with other options such as vibration, thermoelectric, or radio frequency methods [39]. However, these research efforts have shown that even when using power harvesting mechanisms and low sampling rates with measurements once every several hours, a long lasting WSN node with attached gas sensors is hard to achieve. The use of larger energy harvesting elements results in validating these approaches over a span of several hours or a few days only. In contrast, the monitoring system we have presented in this paper has been operating unattended for one month and is still working at the time of writing this paper. Furthermore, these approaches are mainly based on off-the-shelf platforms such as “Waspmote” or the XBee communication module, whereas the research in this work is based on a device that has been entirely developed by the authors. The following section presents our proposed solution for a scalable low-cost monitoring system, with an emphasis on the operation and optimization of BLE air quality beacons.

## 3. Proposed Solution Based on BLE

**Figure 1:**
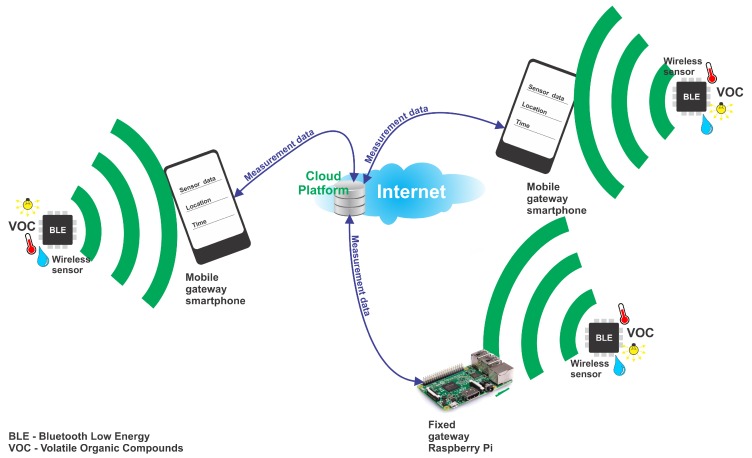
Air pollution monitoring system architecture.

In this work, we focus on the analysis and development of strategies for power usage optimization with BLE beacons (powered by solar cells) able to measure the light intensity, temperature, relative humidity and eCO_2_ and TVOC concentration levels in the air. Figure 1 shows the architecture of the proposed monitoring system where the energy harvesting beacons can be used efficiently. The wireless sensors in the system periodically sample the attached sensors at periods determined by the current light intensity and by the available power and advertise the acquired data. At their turn, observer applications running on platforms such as smartphones or the Raspberry Pi single-board computer that support Bluetooth Low Energy forward the advertisement data to a central server on the Internet, where, combined with location and time information, it can be posted on a real-time air pollution map.

We selected Bluetooth Low Energy because of its low power consumption and because of the low costs of the associated hardware. Furthermore, Bluetooth is a native technology for almost all portable computers and smartphones, making it a good candidate for implementing IoT scenarios that make use of opportunistic and participatory sensing. These Bluetooth compatible electronic systems can be used as relays, gathering the data advertised by BLE sensors and forwarding it to the cloud, where it can be stored and further processed, and where the users can visualize it. The announcement of Bluetooth Mesh Networking [41] and the development of low power chips such as the CYW43012 from Cypress Semiconductor [42] and the ones belonging to the WiLink^TM^8 family from Texas Instruments [43] including WiFi and BLE subsystems will make this technology even more appealing for IoT applications. Furthermore, the release of Bluetooth 5.0 offers significant enhancements as compared to earlier versions of the protocol, and will increase its attractiveness for use in future IoT-based solutions [44,45,46].

### 3.1. Air Quality Sensor Hardware

**Figure 2:**
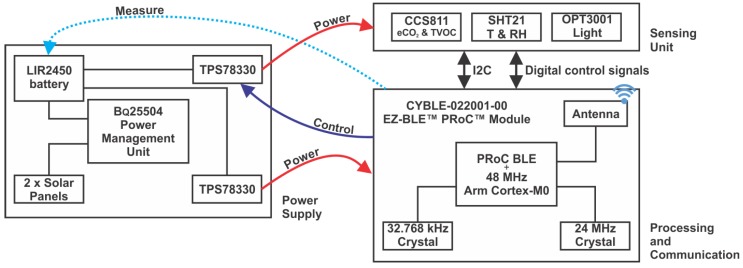
Air quality sensor block diagram.

The core of the developed environmental sensor sensor is the CYBLE-022001-00 communication module supporting BLE [47]. It includes crystal oscillators, antenna, passive components, and a 32-bit microcontroller unit subsystem consisting of a 48 MHz Arm Cortex-M0 CPU with on-board memory. This component controls the operation of the sensor, estimates the available energy, samples the attached sensors at time intervals depending on the computed value, and advertises the acquired data every 5 s. Figure 2 presents the block diagram of the sensor node developed.

The sensing component includes the SHT21 temperature and relative humidity sensor, the CCS811 air quality sensor and the OPT3001 digital ambient light sensor. All of the sensors communicate with the processing unit through an I2C bus. To minimize energy consumption, the three attached sensors use a separate power supply that can be turned on by the main microcontroller only when taking measurements.

The supply unit of the sensor is composed of two high efficiency IXOLAR^TM^SolarBIT solar panels [48] that charge a 3.6 V lithium-ion battery (accumulator) with a capacity of 120 mAh. The solar panels are made up from monocrystalline solar cells and are connected in series. A BQ25504 chip [49] acquires and manages the energy harvested by the solar cells and charges the accumulator in the system. This component, as well as the others included in the design, are targeted towards systems with tight energy requirements such as wireless sensors in WSNs.

#### Power Budget Estimation Mechanism

**Figure 3:**
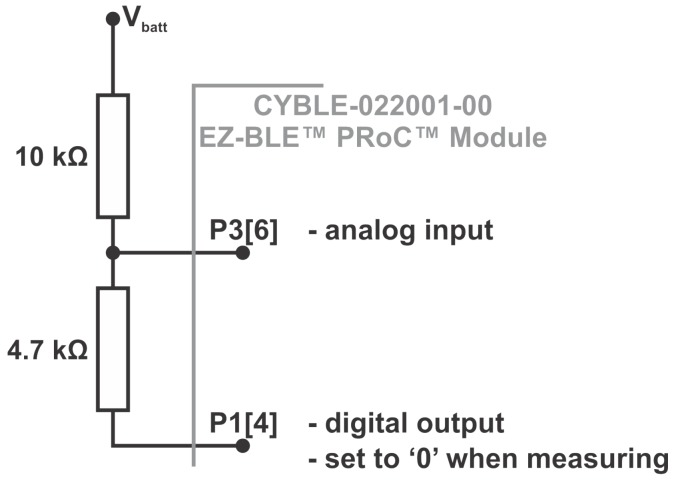
Battery level measurement circuit.

The design includes a simple circuit for measuring the charging state of the battery powering the system that, when combined with other relevant data such as the current light intensity can be used for making decisions on the sampling rate of the attached sensors. The circuit used for measuring the battery level is presented in Figure 3. It consists of a simple resistive voltage divider that provides a satisfactory estimation of the charging level of the attached accumulator powering the system.

The voltage input (P3[6]) is connected to the input of an analog-to-digital converter that is active during the wake up periods of the processing unit. Pin P1[4] of the CYBLE-022001-00 module is set as a digital output with value ‘0’ during voltage measurement and as a high impedance input during sleep. The equation for computing the voltage at the processing unit’s input is:

(1) $$V_{P3[6]}=\frac{4.7\;k\Omega }{10\;k\Omega +4.7\;k\Omega }\cdot V_{battery}$$

**Figure 4:**
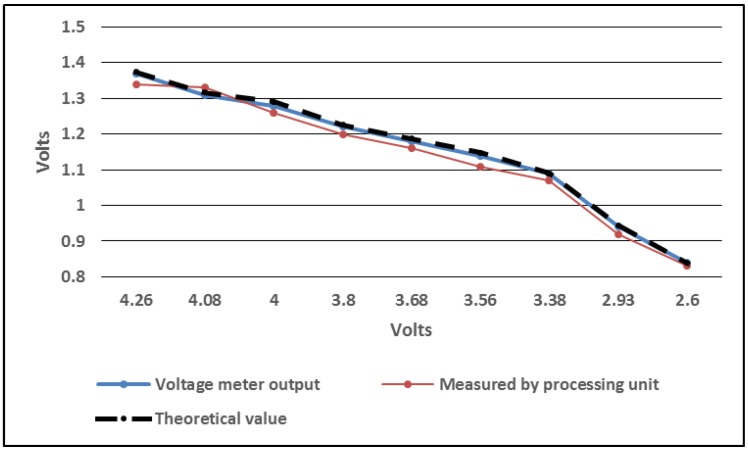
Battery level measurement.

We conducted experiments to determine the relationship between the battery level and the values acquired by the processing unit and to validate the correct values that are used for estimating the available energy budget. The wireless sensor was powered by a laboratory power source and a voltage meter was used to measure the value on the P[3] input during the measurements. Figure 4 presents the data obtained during the experiments. These values are used for estimating the battery level at certain times during the operation of the device. The results show that the method for estimating the charging state of the battery has an acceptable accuracy and can be used for developing strategies for sampling the sensors at different time intervals depending on their power consumption and mode of operation.

### 3.2. Air Quality Sensors’ Firmware

The firmware implemented for achieving low-power consumption during the operation of the sensor device considers that this is not a typical wireless sensor application. In general for wireless sensors operating in WSNs, it is assumed that communication is the operation that requires the largest amount of energy. However, this is not true for beacons or for systems where gas concentrations are measured. This is because here, in general, the sensors are power hungry and might require more energy than the RF transceiver. This is also the case in this work, where the CCS811 sensor consumes more power and requires longer wake-up times than both the other attached sensors and the communication module that is in charge of broadcasting BLE packets.

The application running on the CYBLE-022001-00 module implements a BLE generic access profile (GAP) broadcaster that advertises the data acquired from the attached sensors in non-connectable undirected mode on all channels with an advertising interval of 5 s Since the sensors consume more power than advertising, they are sampled at different periods, depending on their power consumption and on the voltage level estimated for the rechargeable battery and on the light intensity. Therefore, the SHT21 temperature and relative humidity and the OPT3001 light intensity sensors can be powered and interrogated every minute. At this time, the battery voltage is also computed by sampling the internal ADC and a scaling factor of 3.14 is applied. This scaling represents the ratio between the real voltage of the battery and the measurement of the voltage on the output of the resistive voltage divider as shown in Figure 4. If the illumination and the battery voltage are above certain thresholds, as the following paragraphs will show, the active period is prolonged and the attached gas sensor is also interrogated. Between these activities, the device is programmed to enter low-power mode in order to save as much energy as possible.

The main operations performed by the MCU are listed in Algorithm 1. This is a simplified flow with the actual application being tailored based on the requirements of each sensor in the system. We present a more detailed view of the firmware that was implemented and the experiments conducted in the following sections. Different periods were selected for taking measurements to reach a satisfactory compromise between power consumption and data accuracy.
**Algorithm 1** Wireless sensor application main activities1:Initialize components2:Set sensors parameters3:**while***True***do**                          ▹ Main app. loop4:  Process BLE events5:  **if** (1 min. passed) **then**6:   **if** (batt. level or light data from RAM > thresholds) **then**  ▹ From prev. active period7:    Power up sensors8:    Wake up all components ▹ Can be executed even if device was not in low power mode9:    Measure T and RH, save data to RAM10:    Get light intensity, save data to RAM11:    Perform gas measurement, save data to RAM12:    Put all components in sleep mode13:    Power down sensors14:   **end if**15:   Get batt. level, save data to RAM16:  **end if**17:  Update advertising payload with data from RAM18: Enter low power mode19:**end while**


### 3.3. Communication between the System’s Components

The developed system uses advertisement packets provided by the Bluetooth Low Energy networking technology. Therefore, the device used for measuring the environmental parameters represents a BLE beacon. Advertising is one of the two modes of communication using Bluetooth besides data transmission after a connection is established, and has been widely used for tracking applications [50] and sensor systems [51]. This mechanism implies that a BLE peripheral, represented in our case by the power-harvesting sensor node, transmits packets on channels 37, 38, and 39 of the 2.4 GHz spectrum used by Bluetooth, that will be received by any nearby scanning Central device.

As in other BLE beacons, such as Apple’s iBeacon, the variable data representing the temperature, relative humidity, light intensity, air quality related information, and available energy indicator, are included in the Manufacturer Specific Data field of the advertisement packets. The dynamic payload also includes a field indicating the number of minutes since the last interrogation of the air quality sensor, so that the receiver of the message can add the correct timestamp to the acquired information. Thus, bytes 16 and 17 contain the temperature, 18 and 19 hold the relative humidity, 20 and 21 hold the voltage, and are followed by the light intensity which also takes 2 bytes. The number of minutes since the recording of eCO_2_ and TVOC levels is placed in the 24th byte, while the air quality related data is placed between the 25th byte and the 28th byte.

There are other solutions that are based on this type of communication. These include the CYALKIT-E02 Solar-Powered BLE Sensor Beacon from Cypress Semiconductor, or the Environment Sensor 2JCIE-BL01 from Omron. However, in this we focus on the energy efficiency of power-harvesting beacons that incorporate air quality sensors such as the CCS811.

### 3.4. Gateway Implementation

A Raspberry Pi single-board computer was used for acquiring data from several beacons and for validating the strategies used for optimizing their energy consumption. The application running on the RPi embedded platform makes use of several python scripts whose main task include the observation and processing of non-connectable advertisement packets (Algorithm 2). The address of each intercepted packet’s sender is compared with a list of known LE Bluetooth device addresses. If a packet that was sent by a known peripheral is received, then the payload is parsed and the data is saved into a SQLite database, along with the device address and the current time. From the gateways, the information can be sent at a central station, where it can be used for generating reports or fine grained environmental parameters and air pollution maps, showing historical data and current trends.

During the experiments, the data gathered by a single gateway from different BLE beacons was stored and displayed on line charts. However, other devices such as smart phones could be used for gathering data from beacons placed at different locations in zones of interest in order to attach the appropriate time and location information to the data and finally relaying it to the cloud. This participatory sensing model could help achieve fine-grained maps of environmental conditions and pollution in crowded areas or even in entire cities.
**Algorithm 2** Observer application1:**if** (no database) **then**2:  Create database3:**end if**4:Connect to the database5:**while***True***do**                          ▹ Main app. loop6:  Scan for BLE advertisements7:  **if** (BLE advertisement from known address) **then**8:   Process manufacturer specific data9:   Apply timestamp and save to database10:  **end if**11:**end while**


Section 4 presents the power consumption of the BLE beacons developed, and the circuit that was implemented for providing them with on-board battery level measurement capability. In this section, we also present the way in which the wireless sensor takes advantage of the values measured and on its current power supply state for achieving energy autonomy.

## 4. Power Consumption

### 4.1. Power Consumption Measurement Using Laboratory Equipment

**Figure 5:**
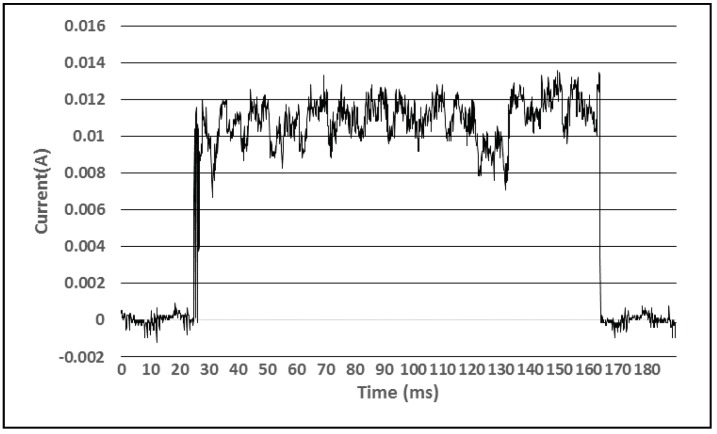
Consumption profile during one active interval (T, RH, light, and battery level measurement).

To analyze the power consumption of the sensor devices and to estimate their operation period using the complete charge of the accumulator we used a Tektronix TDS2012C oscilloscope and a special setup, consisting of an INA138 integrated circuit and a resistance [21]. This was set to measure the power consumption profile of the BLE environmental beacons depending on the activities they perform. Figure 5 presents the power consumption of a BLE sensor that measures the temperature and relative humidity, light intensity and battery levels during one active interval, lasting for 140 milliseconds. This event consumes a total energy of 20.51 J when taking place once per second. The accumulator contains 1600 J of energy, so the device could operate for 80 h on a single charge if the temperature and relative humidity and light intensity sensors are sampled once per minute.

**Figure 6:**
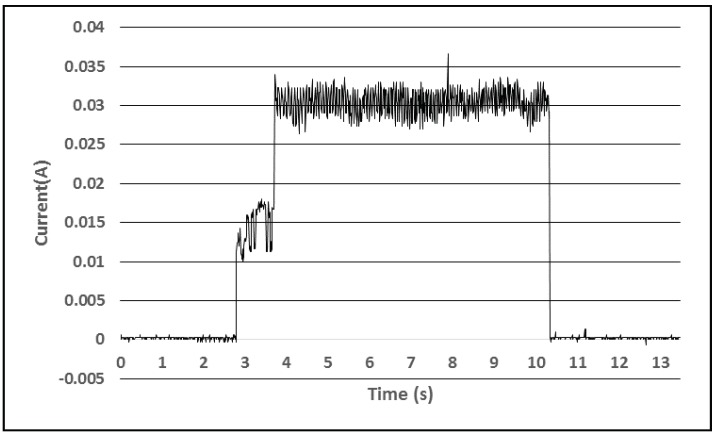
Consumption profile during one active interval (T, RH, light, battery level, and air quality parameters measurement).

Figure 6 shows the power consumption of a wireless sensor that also measures the eCO_2_ and TVOC values.

During the experiments it was observed that the CCS811 air quality sensor needs a warm up period of several seconds before performing this measurement when it is powered up and then is set to take one measurement each second. The active period in this case was 7.6 s. In the case in which the sensor interrogates all the attached sensors (SHT21, OPT3001, and CCS811) once per minute, 50.62 J of energy are necessary. Therefore, if only this event would take place every minute, the power supply would get depleted in 32 h if the accumulator is not charged during this period. However, for the experiments longer periods were set for the sampling rate of the air quality sensor (in the order of tens of minutes) because in this way the accumulator can recover by being charged by the energy harvesting module.

**Figure 7:**
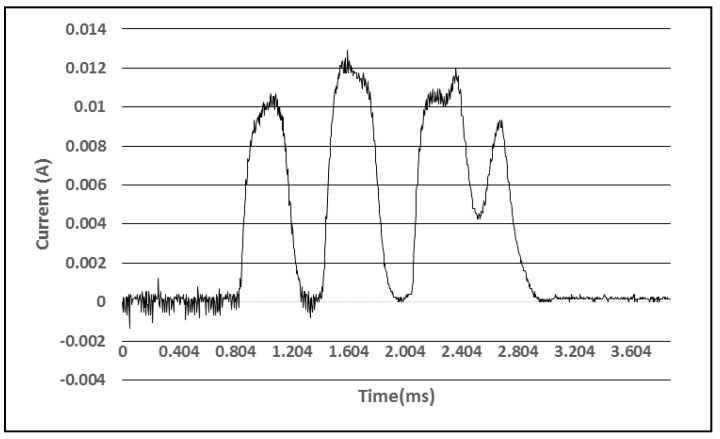
Consumption profile during advertising.

Finally, the power consumption during the transmission of a single advertisement packet was measured. As can be seen in Figure 7, the transmission of such a packet requires 2.1 milliseconds to complete and a total energy of 0.14 J if the advertising interval is set to one second. Thus, 11,500 advertisement packets can be sent on a single accumulator charge. The average power consumption of the device in sleep mode is 0.4 J per hour and all the energy stored in the power supply would allow the operation for 5 months in sleep mode.

By knowing the energy requirements of the environmental wireless sensor during all the activities it performs, an estimation of the operation period using a single accumulator charge can be computed. Based on the values presented in the figures above, the periods obtained are of roughly 72 h for a device that does not read the air quality related parameters and roughly 30 h for one that samples also the CCS811 sensor every minute. This leads to the conclusion that the accumulator has to be charged as efficiently as possible between the active periods of the sensing system. However, because solar cells are used, the charging operation is highly dependent on weather conditions, the efficiency dropping substantially during cloudy periods. Furthermore, the device is not able to charge the accumulator during the night. Therefore, efficient self-adaptive mechanisms that choose the proper moments for performing a measurement have to be devised, as the following subsection will show.

**Figure 8:**
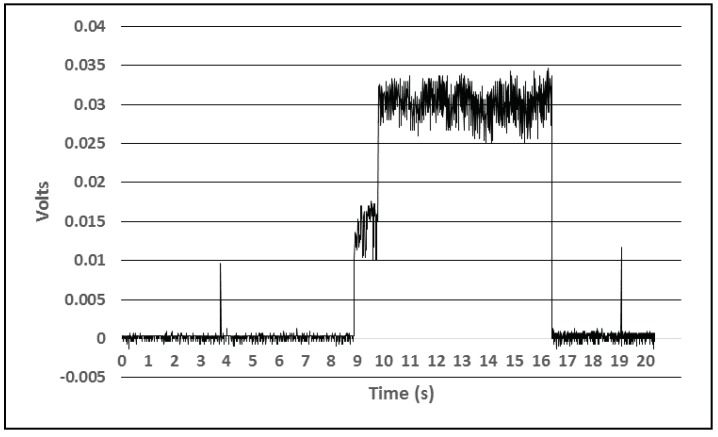
Consumption profile during advertizing and measurement.

Figure 8 presents the power profile of the device during normal operation when a measurement takes place and advertisement packets are sent. Here, it can be seen that an advertisement (short duration pulses on the chart) requires much less energy than the interrogation of the attached sensors (action taking place between the 8th second and the 16th second).

### 4.2. Self-Adaptive Operation Based on on-Board Measurements

The environmental sensor can measure the current charging level of the accumulator and the current light intensity. As a result, it can take the decision whether to sample the CCS811 sensor during the current active period, or postpone it for an active period in the future. When a measurement is missed due to the low level of the power supply, a counter is incremented and saved in the memory of the device and in the advertisement packet, so the observer application knows to attach the proper timestamp to the data. For example, if the power supply level is low, but the light intensity recorded is high (above 50,000 LUX, for example, while 111,000 LUX represent bright sunlight), a measurement can take place. As a result, the device adapts the measurement rate depending on the light intensity and on the current power supply state. Algorithm 3 lists the activities performed by a wireless sensor that adapts the sampling period of the sensors depending on the power supply level and the light intensity.

The experimental results section presents the data acquired by the BLE beacons we have developed as a proof of concept system when different parameters for the adaptive scheduling actions and sensor configurations are used.
**Algorithm 3** Wireless sensor application (T, RH, light intensity, eCO_2_ and TVOC)1:Initialize components2:Test CCS811 operation3:Get batt. level4:**while***True***do**                          ▹ Main app. loop5:  Process BLE events6:  **if** (1 min. passed) **then**7:   Power up sensors8:   Wake up all components9:   Measure T and RH, save data to RAM10:   Set light sensor for continuous operation11:   Wait for light conversion12:   Get light intensity, save data to RAM13:   **if** (batt. level and light data from RAM > thresholds) **then**14:    Set mode for gas sensor15:    Start gas measurement16:    Save gas sensor output to RAM17:   **end if**18:   Get batt. level, save data to RAM19:   Compute thresholds values20:  **end if**21:  Update advertising payload with data from RAM22:  Enter low power mode23:**end while**


## 5. Experimental Results

**Figure 9:**
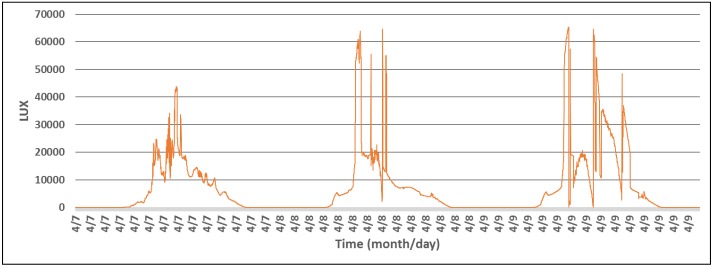
Light intensity measured by a T, RH, and LUX beacon during three days.

**Figure 10:**
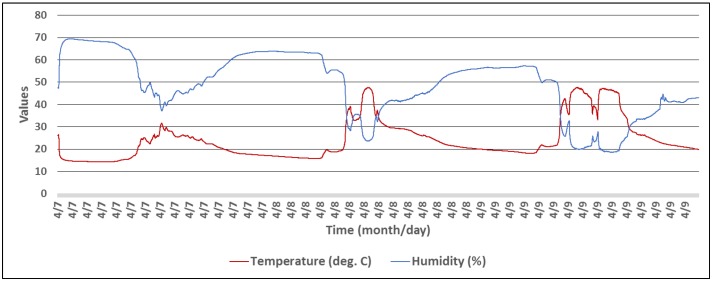
Temperature and relative humidity for a T, RH, and LUX beacon during three days.

For the first set of experiments, a wireless sensor was set to measure T, RH, and light intensity levels, during a period of three days. The experimental setup consisted of the wireless sensor, that was placed in an area exposed to natural light during the day, and a Raspberry Pi single-board computer running the Observer application. The activities performed by the sensor are presented in Algorithm 4, and the collected data is presented in Figure 9 and Figure 10.
**Algorithm 4** Wireless sensor measuring T, RH, and light intensity1:Initialize components2:Get batt. level3:**while***True***do**                          ▹ Main app. loop4:  Process BLE events5:  **if** (1 min. passed) **then**6:   **if** (batt. level and light data from RAM > thresholds) **then**7:    Power up sensors8:    Wake up all components9:    Measure T and RH, save data to RAM10:    Get light intensity, save data to RAM11:   **else**12:    Increase postponed measurement counter13:   **end if**14:   Get batt. level, save data to RAM15:   Power down all sensors16:   Prepare for low power mode17:  **end if**18: Update advertising payload with data from RAM19:  Enter low power mode20:**end while**


**Figure 11:**
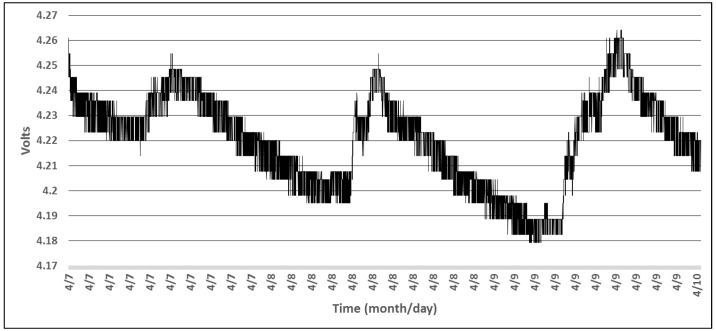
Accumulator level for a T, RH, and LUX beacon during three days.

Figure 11 shows the battery level during all the time when the wireless sensor was operating thereby revealing that the accumulator is actually charged when a one minute sensor sampling interval is used and the solar cells are exposed to direct sunlight during the day. From Figure 11 we note that the acquisition of the battery level is not a highly accurate measurement, but the readings can give a satisfactory estimation of the charging state of the accumulator, so that appropriate decisions can be taken by the processing unit with respect to the sampling period of the attached sensors. We attempt to find a compromise between the measuring circuit power consumption, complexity and accuracy. This was achieved by selecting the battery level and light intensity thresholds that lead to the modification of the sampling rate used for the CCS811 sensor, the most power hungry component in the system.

**Figure 12:**
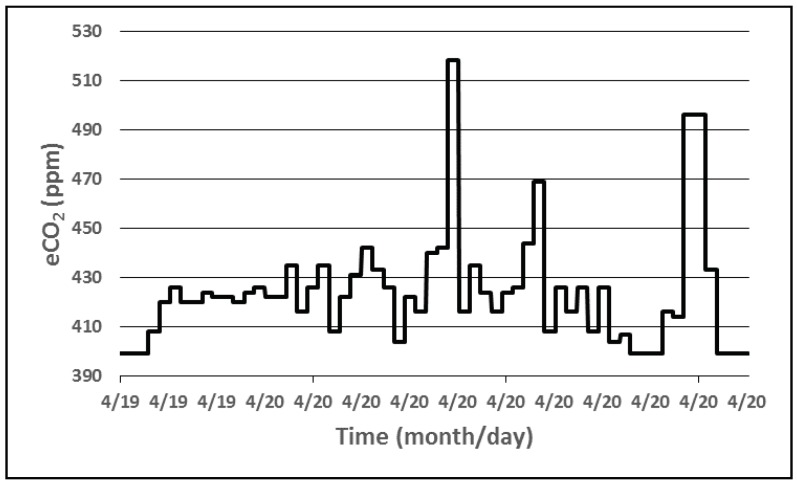
eCO_2_ concentration level measured.

**Figure 13:**
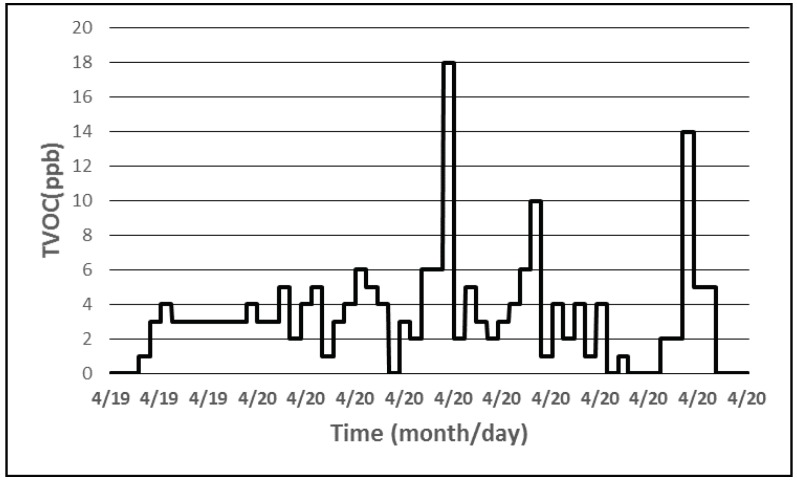
TVOC concentration level measured.

The data gathered over these three days shows that a device that measures the temperature, relative humidity and light intensity, can operate solely on the energy harvested by the solar cells that charges the attached accumulator. The next step was the inclusion of the sampling of the air quality sensor into the application. Therefore, different sequences of events similar to the one listed in Algorithm 3 were implemented in the firmware of the wireless sensor. Figure 12 shows the equivalent CO_2_ concentration measured by a device during a day while Figure 13 represents the TVOC plot.

**Figure 14:**
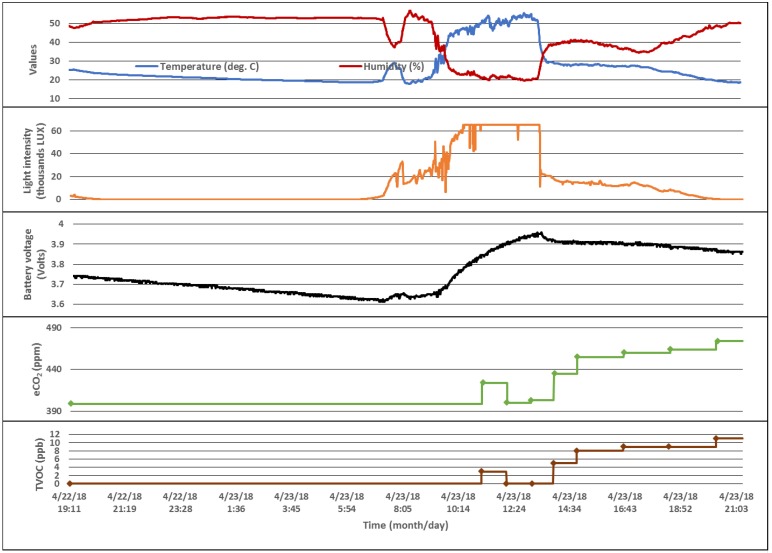
Data acquired by a T, RH, LUX, eCO_2_ and TVOC beacon.

The data acquired by a device that implements an application flow similar to the one presented in Algorithm 3, where the CCS811 sensor is sampled according to different intervals depending on battery level and light intensity, is shown in Figure 14. The points where the eCO_2_ and TVOC values are measured are represented on the plots with diamonds. Since the values are represented on two bytes, the light intensity that is transmitted does not exceed this limit even though the measured value is larger.

**Figure 15:**
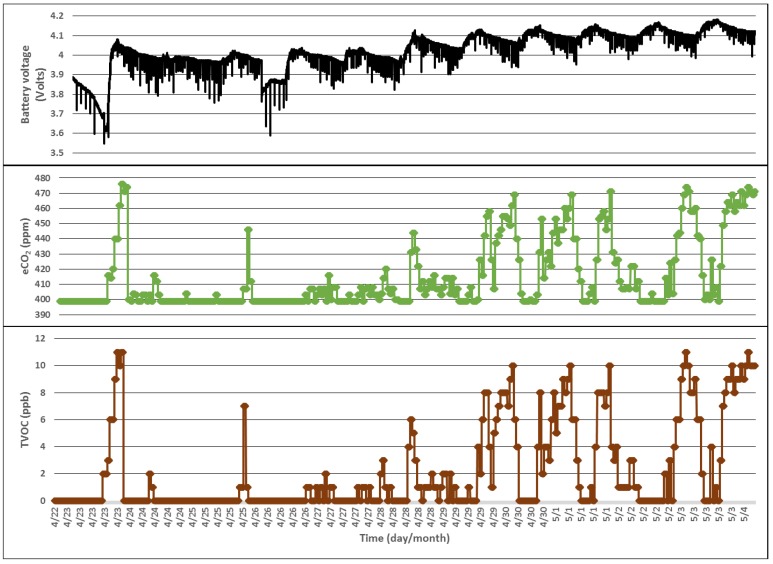
Energy budget and air quality parameters during 12 days.

The results obtained (Figure 14 and Figure 15) demonstrate that with efficient activity planning, rather power hungry sensors or sensors that require special sampling procedures, can be included in energy-autonomous devices, such as wireless sensors based on solar cells. The developed strategies could be combined with artificial intelligence techniques to enable the forecasting of weather conditions and further optimize the power demands of tightly constrained electronic devices.

## 6. Conclusions and Future Work

We analyzed the power requirements of an energy harvesting wireless sensor that uses BLE advertisement packets to transmit the measured temperature, humidity, light intensity, and air quality in this work. This analysis was used for developing self-adaptive strategies for optimizing the power consumption of the sensing device based on the measurement of available energy (current accumulator charging state) and on the harvesting conditions (measured light intensity). The wireless sensor developed was tested for a month in a scenario (as close to a real one,) as part of a proof-of-concept monitoring system that gathers the sensed data, which are subsequently used for generating reports in the form of charts. The results obtained during the experiments demonstrate that energy-efficient autonomous operations of wireless sensors that measure the temperature, relative humidity, light intensity, and equivalent CO_2_ and TVOC levels have been achieved. Furthermore, the sensing beacon developed and analyzed has the advantage of being low-cost (less than 150 Euro) along with a small footprint (35 mm × 35 mm). Therefore, the research demonstrates the feasibility of manufacturing sensors capable of measuring gas concentrations that are powered entirely by small form factor energy harvesting elements that can operate in the context of IoT.

The experimental setup consisted of a proof-of-concept system that represents a complete IoT-based solution for monitoring temperature, relative humidity and equivalent CO_2_ and VOC concentration levels within a large area. By using Bluetooth Low Energy, a technology native to a wide range of personal electronic devices, we argue that our developed monitoring system can make use of participatory and opportunistic sensing. Therefore, the complete monitoring solution presented and analyzed in this work can help achieve real time fine-grained air pollution maps, and can be applied to smart cities or buildings. The major drawback of the proposed solution is that it does not provide measurements as detailed and as accurate as traditional stations. However, this is compensated by the advantage of not requiring a fixed infrastructure, the gateways being represented by BLE-enabled electronic devices, such as smartphones or laptops. In this way, the maintenance activities are less demanding and the costs for commissioning and support are reduced. This means that the wireless sensors this solution incorporates can be deployed in large numbers over large areas and can be used for complementing existing traditional monitoring installations. In this way, fine-grained maps showing spatial data and the changes in environmental conditions and pollutant levels close to the source of emissions can be obtained. This class of monitoring applications also involves the citizens and aims to increase people’s awareness towards the environment by enabling efficient data collection from sensors by personal devices such a smartphones.

Our future work will investigate the inclusion of other air quality sensors within energy-autonomous platforms and the development of cloud applications for gathering the data relayed from the gateways. In the future, we will investigate the use of Bluetooth 5.0 in environmental monitoring applications.
